# Marginalisation, Ebola and Health for All: From Outbreak to Lessons Learned

**DOI:** 10.3390/ijerph16173023

**Published:** 2019-08-21

**Authors:** Clare Shelley-Egan, Jim Dratwa

**Affiliations:** 1 Work Research Institute, Oslo Metropolitan University, PO. Box 4, St. Olavs Plass, NO-0130 Oslo, Norway; 2 Law and Science and Technology Studies (LSTS), Free University of Brussels (VUB), 1050 Brussels, Belgium

**Keywords:** marginalisation, Ebola outbreak, epidemic, crisis, West Africa, global health, lessons learned

## Abstract

The Ebola epidemic in West Africa between 2014 and 2015 was the deadliest since the discovery of the virus four decades ago. With the second-largest outbreak of Ebola virus disease currently raging in the Democratic Republic of the Congo, (DRC) it is clear that lessons from the past can be quickly forgotten—or be incomplete in the first instance. In this article, we seek to understand the health challenges facing marginalised people by elaborating on the multiple dimensions of marginalisation in the case of the West Africa Ebola epidemic. We trace and unpack modes of marginalisation, beginning with the “outbreak narrative” and its main components and go on to examine other framings, including the prioritisation of the present over the past, the positioning of ‘Us versus Them’; and the marginalisation—in responses to the outbreak—of traditional medicine, cultural practices and other practices around farming and hunting. Finally, we reflect on the ‘lessons learned’ framing, highlighting what is included and what is left out. In conclusion, we stress the need to acknowledge—and be responsive to—the ethical, normative framings of such marginalisation.

## 1. Introduction, Materials and Methods

The Ebola outbreak in West Africa between 2014 and 2015 was the deadliest since the discovery of the virus. Almost 30,000 people were affected by the disease, with tragic consequences for individuals who lost family members and livelihoods, survivors left with a legacy of stigma and for community fabrics [1]. A slew of “lessons learned” articles aimed to distil the hard lessons of the catastrophe in order to mitigate another one. Yet the current Ebola epidemic in the Democratic Republic of the Congo (DRC) reminds us that lessons from the past can be too quickly forgotten—or were incomplete in the first instance. 

Four decades after the initial identification of the Ebola virus and five years after the dangers and disastrous consequences of Ebola were writ large in West Africa; and it is not clear that the lessons have been the right ones. As the successful treatment of cases in Europe and the US during the 2014–2015 outbreak has shown, victims of Ebola are not necessarily consigned to rapid and painful death. Indeed, with the right medical treatment and quarantine conditions, Ebola is not a guaranteed death sentence. Why, then, does it remain so difficult to go beyond a rote medical response and repetitive media narratives of “mistrust” in doctors and science? In this paper, we seek to understand the health challenges facing marginalised people by explicating the multiple dimensions of marginalisation in the case of the Ebola epidemic of 2014–2015. 

But what is marginalisation to start with? In healthcare, populations or social groups with lower health status—associated with social, environmental, biological and/or political determinants of health—are frequently termed ‘marginalised’, ‘vulnerable’ or ‘underserved’. There are numerous tensions between these notions in the literature. Each of them is recurrently contested (for example, see Reference [2,3]), as are the connections between them (for example, see Reference [4]).

As regards marginalisation, Lynam and Cowley [5] have provided a seminal delineation of marginalisation as a sense of being overlooked, categorised or misrepresented, emphasising societal isolation of the marginalised as ‘other’ (which also helps to clarify how marginalisation is related to, but different, from vulnerability); however, tensions abound as to definitions, as well as operationalisations [6], including in relation to ‘social determinants of health’ [7].

Furthermore, each discipline has its own conceptual map and scholarly genealogy of this notion. For example, in the nursing science literature, marginalisation was proposed as a nursing theory by Hall et al. in 1994 [8,9,10]. It then underwent a progressive transformation which led to the inclusion of additional elements, such as Eurocentrism [11] and then went through a series of expansions integrating scholarship on globalisation, privilege and intersectionality [12]. The theory of marginalisation itself has had to open and expand its own margins. Such is the work that we propose in this paper.

Informed and inspired by this diverse lineage, the present article examines the processes and practices through which marginalisation occurs, mobilising the literature on anthropology of the Ebola virus disease, development studies, politics and international relations of sub-Saharan Africa, medical ethics, bioethics, and global health. 

This paper sheds light on the health challenges facing marginalised people by explicating the different forms of marginalisation in the case of the Ebola ‘outbreak’ of 2014–2015. Indeed, the very notion of ‘outbreak’ is problematic. The concept of ‘outbreak’ indicates and performs a process of demarcation; between the central and the peripheral, between the normalised (or privileged) and the marginalised, between the settled and the unsettling, between what is to be kept safe and what is to be kept at bay and confined—with the risk that the latter will at times ‘break out’. These demarcations pertain to individuals and groups, but also to ways of knowing, of making sense, of acting in the world. In other words, in each case, with each demarcation, it is not only people who are marginalised, but also worldviews, epistemologies, or forms of life. 

We trace and unpack these modes of marginalisation, beginning with the “outbreak narrative” and its main components, propagated—as a pandemic in its own right—by the international media. We then consider the present versus other temporalities, that is, the dominance of the ‘now’ and the ‘urgent’ over the deeply entrenched and neglected (i.e., over pre-existing, structural and institutionalised conditions that serve to exacerbate epidemics and their containment). Another key form of marginalisation is the ‘Us versus Them’ framing, notably in the ‘north versus south’ configuration. This demarcation—with ‘Us’ at the top, at the centre, and ‘Them’ at the periphery, at the margin—takes the shape of situations of inequality and selective caring. Another manner of ‘Us versus Them’ is visible in the low level of trust in government and health authorities and in the reference to Ebola as “an African disease spread through African culture” [13]. This leads to the examination of cognate marginalisation, science versus other practices and forms of knowledge, before we turn to practices concerning life and death, i.e., funerals and burials, as well as farming and hunting. Progressing through this succession of marginalisations, the paper, thus, proceeds through a series of reframing and de-centring moves (unpacking those frames, opening those black boxes, endeavouring to shed light on what is left—or placed—in the dark). The last of those frames is that of the ‘lessons learned’, which risks solidifying the partition between what is upheld as most relevant and what is left confined to the margins, and which the paper undertakes to explicate. This goes to the paper’s own ‘lessons learned’ as it concludes with a summary of its main steps and achievements.

## 2. Results and Discussion

### 2.1. The ‘Outbreak Narrative’

The Ebola outbreak in 2014 and 2015—which began late in December 2013 and ended during January 2016—in the Mano River Region of West Africa was unprecedented in scope and severity, affecting three of the poorest countries in the world. The outbreak was the deadliest occurrence of the disease since its discovery in 1975, with 28, 616 cases of the disease and 11,310 deaths [14]. The outbreak has led to more cases and fatalities than the aggregated total of all the former 20 outbreaks [15]. Other, grave consequences of the Ebola outbreak included the neglect of non-communicable diseases, such as malaria, loss of livelihoods, due to disruption of food supplies and trade routes, thousands of orphaned children left behind, and the stigmatisation of sufferers and relatives (especially of children) [1,16]. Survivors of the virus face the possibility of an array of unpleasant and challenging conditions, including vision loss and conjunctivitis, joint and muscular pain, fatigue and headaches, not to mention psychological problems, such as depression, survivor guilt, and post-traumatic stress disorder [17]. 

Prior to this particular Ebola outbreak, West Africa had no recorded Ebola deaths [18]. Moreover, Ebola had been concentrated in rural areas, where the public health response was rapid enough to prevent spread to populated cities. An Ebola outbreak in urban populations in Africa was unprecedented. In April 2014, the virus emerged in the capitals Conakry (Guinea), Freetown (Sierra Leone) and Monrovia (Liberia) [15]. Furthermore, the virus spread across international borders, with cases linked to the outbreak reported in Mali, Senegal, Nigeria, Spain, the United States, and the United Kingdom [19].

Very little happened internationally until the disease began to fulfil what has been termed the “outbreak narrative” [20,21], as read by the globally powerful North. This story involves the emergence of a disease in a remote location that goes on to spread across a world highly connected through globalisation to threaten “us all”, or specifically, the global North [21]. It is this narrative that—fuelled by a handful of cases in Europe and in the U.S. and concern about protecting the west—finally moved publics and politicians to launch a large—scale international, militarised response and belated investment in vaccines and experimental treatments [1,22]. The Ebola “outbreak narrative” emphasised local-level poverty and cultural and ecological practices as the major factors of causation in the epidemic [23]. 

The incorporation of the factors just mentioned in the outbreak narrative painted “(…) an overly simplified and often false picture (…)”, and laid “(…) primary blame for the crisis at the feet of those most tragically affected” [23]. Moreover, the narrative excluded consideration of the personal tragedies and losses of those people affected; instead, those regions and populations mostly affected by the disease were consigned to secondary characters in a narrative about the West and the potential risks and dangers implied by the spread of the disease across international borders [22]. 

This familiar—but highly misleading—narrative of causation omitted multiple other factors in the causation of the epidemic, ranging from the effect of rapid urbanisation with inadequate basic infrastructure, entrenched poverty and the detrimental implications of a lack of care and proper governance following post-war conflict [1,13,21,24,25,26,27]. While there is *some* truth in this narrative, the reduction of the factors informing and exacerbating this catastrophic outbreak to this framing obscures fundamental political, cultural, historical and ecological determinants of the outbreak. Moreover, the storylines of the narrative both marginalise—and are in direct conflict with—the actual, lived realities of people affected [1,22,28]. It is precisely these omitted factors, and with them the neglected strands and lives, that we bring back to the fore in the following sections of the paper. 

We turn first to reflect on the tension between the framing of the outbreak as ‘new’ and ‘emerging’ versus *de facto* structural, normalised and institutionalised dimensions of impoverishment and neglect and their bearing on the containment of the outbreak. 

### 2.2. The Present Versus Other Temporalities

There have been 29 outbreaks or case reports of Ebola virus reported since the virus was first identified in 1976. These outbreaks and cases occurred in rural communities in Sudan, the Republic of Congo, Gabon, Uganda and the Democratic Republic of Congo (DRC) and were small in size, with seven outbreaks involving more than 100 cases [29]. The West African outbreak was the first outbreak to lead to a major global public health threat and was unprecedented in the spread of the virus across international borders [29]. Notwithstanding the novel features of this outbreak, the challenge of addressing the epidemic was not a new or unprecedented challenge [22,30,31,32]. Similarly, as we have suggested elsewhere [33], in times of emergent health crises, irresponsibilities may arise in the manner in which responses are framed and implemented. Specifically, this relates both to the way in which “such situations emerge under a crisis frame, and to pre-existing irresponsibilities which condition how such crises unfold” [33]. Care for the future, we argue, necessitates attention to ongoing inequalities, which gain particular salience in times of emergency and which condition how crises are managed. 

The lack of preparedness of the three countries to tackle the Ebola outbreak can be explained by two primary factors. One major factor was the consequence of post-war fragility and unstable leadership (in the case of Sierra Leone and Liberia) [15]. This post-war situation has brought other serious consequences to the fore, such as a pervasive lack of trust in both government institutions and the medical system which has served to undermine responses to the crisis further [34]. In the case of Ebola, as Wilkinson and Leach observe, “(…) governments that have long been experienced as deeply detached from their publics, and perceived not to act in their interests, are now the enforcers of biomedicine at its most authoritative” [21]. For example, in Liberia and Sierra Leone, militarised and heavy-handed responses in the form of quarantines, roadblocks and curfews brought back war-time memories and further chipped away at trust in public authorities [1,16]. We shall return to the issue of resistance to infection control measures in Section 2.3. 

The second major factor contributing to the lack of preparedness is the lack of investment over decades in core public health infrastructure and services. Colonial occupation and exploitation of the three countries have left them poor and impoverished. Further impoverishment can be traced directly back to sparse spending on public services, as a result of the International Monetary Fund (IMF) conditions [15,26]. The three countries have been subject to IMF Structural Adjustment Programmes which, under the “macro-economic stability” paradigm have curtailed public spending in health systems and led to the erosion of necessary physical and social infrastructures over decades [15,24]. Moreover, IMF loan conditions have placed a limit on public sector wages, including those of health care workers, serving to decentralise health care and undermine the mobilisation of centralised and coordinated responses to outbreaks [19]. 

These factors, along with the impact of political instability, led to run-down facilities, insufficient numbers of health personnel and demoralising working conditions [15]. Even before the crisis inflicted a heavy toll on health workers, the three countries were grossly under-resourced. For instance, Liberia had just 57 doctors and midwives in 2008, while Sierra Leone only had 136 doctors and 1017 nurses [15]. In addition, as Sanders et al. [26] note, the ‘brain drain’ phenomenon has meant that these countries “subsidize(*sic*) the health systems of rich countries”, making it extremely challenging for countries in the region to establish their own, credible health systems (p. 648). 

Containment is the major challenge of Ebola and includes the isolation of suspected Ebola cases, infection control and universal precautions, contact tracing and monitoring, surveillance and enhanced awareness in local communities and internationally [35]. The “broken health systems” found in the affected countries meant that being treated, or working, in a hospital was extremely hazardous. In the absence of trained staff, isolation units, personal protective equipment, and strict infection control, hospitals became “amplification” points for the spread of the virus, putting healthcare workers at significant risk [15,26]. One tragic irony here is that Liberia is home to the world’s largest natural rubber producing operation, mainly focused on producing rubber for car tyres, but also for companies manufacturing medical components, including the latex gloves, lacking in health facilities in the region [36]. 

On learning that hospitals were unsafe and rife with Ebola contamination, patients with Ebola-type symptoms stayed away [37]. As a result, patients with other health conditions—including AIDs, malaria, heart disease and cancer—also stayed away, suffering or dying prematurely from a lack of care [37]. Tragically, many of the region’s recent gains, including a sharp reduction in child mortality, were undone, largely due to the fact that basic medical services have been shut down as a result of the crisis [38]. While efforts were made to establish a broader network of secure, well-run isolated units, accessibility remained an issue, with long distances, inaccessibility of villages, and inability to pay for modes of transport, such as hammocks, factors for those affected [39].

Population mobility in the region comprised another critical challenge for containment and was a key contributing factor in the rapid spread of the virus. Human movement in the West African region is considered a particular feature of the region, with migration rates exceeding movement in the rest of the world by more than seven-fold [40]. Such large-scale population movement in the region has come about as a result of decades of conflict and the search for greater socioeconomic conditions. Another major factor in the rapid build-up of the disease in its epicentre was the “cross-border family-based and market networking among Kissi-speaking settlements divided by colonial boundaries into three separate states (Guinea, Liberia and Sierra Leone)” [40]. 

Notwithstanding the link between entrenched and structural inequalities and the severity of the Ebola crisis, media commentators, the general public, and international agencies largely ignored questions regarding the political-economic drivers of poverty in the region, leading to a sort of ‘Us versus Them’ positioning. 

### 2.3. ‘Us versus Them’, ‘North Versus South’

One key aspect of the ‘Us versus Them’ positioning is the framing of the Ebola virus disease as an African disease spread through African culture [13,24]. As Li and Jones [13] observe “African political, social, and economic context is taken as a given, set aside in a “black box” and untouched by outbreak control efforts. African “Otherness” overpowers the possibility of a non-cultural causality in the dominant discourse, and other factors are left unexamined as potentially causal or exacerbating” [13]. The ‘othering’ of Ebola to Africa over the past decades has “reinforced the virus’s mystique by squarely associating it with far-flung places out of the geographic purview of all but the most adventurous virus-hunters” [32]. This is despite the fact that Ebola is not endemic to any particular place on the African continent [32]. Furthermore, this is also despite the fact that culture has not proven to be a barrier to containment in Africa in the past. In this particular Ebola outbreak, the Nigerian health system, for instance, was able to curtail the spread through the management of the disease through contact tracing, monitoring and isolation of cases [41].

Another element of ‘Us versus Them’ that quickly became evident as the crisis unfolded was one of inequality and selective caring. The deadly and fast-moving nature of the virus in the West Africa outbreak led to a shift in focus from public health measures to prophylactic measures to treat infected individuals [42]. An unapproved experimental drug called ZMapp was administered to two US aid workers and a Spanish priest in August 2014, all of whom were evacuated out of the region, as were health personnel from the United Kingdom and the Netherlands. As Donovan (2014) observes, the world and the region had little knowledge of this unapproved medication and the limited doses available until this particular moment [34]. Furthermore, by early October 2014, it was reported that 337 local health workers had contracted Ebola and 181 had died, while there had been no evacuation of local health workers [26]. Sierra Leone’s top Ebola doctor, Dr. Sheik Humarr Khan, died of the disease in late July 2014, while being considered for evacuation to a European country, and while a dose of Zmapp was available. The WHO refused to allow another infected doctor in Sierra Leone, Dr. Olivet Buck, to leave the country or to fund an evacuation, saying that it would work to provide Buck with “the best care possible” in Sierra Leone [26]. Dr. Buck died during September 2014. However justifiable, the concern and relief about the evacuation and treatment of western healthcare personnel nonetheless highlights the socially divisive “optics of racial preferences” [37] and raised questions about how the world responds to the Ebola crisis and to those working on the ground to stop its spread [26]. 

A glaring consequence of this sort of process of ‘Othering’ was the loss of the humanity of the Ebola victims. Raphael Frankfurter [43] illustrates this loss of empathy and compassion in the following anecdote concerning reported tensions between victims and healthcare workers: “As tensions between health workers and patients have gotten more heated, discussions of the disease have begun to take into account social context. ‘You may not be able to walk in and just say ‘OK, who in this village has Ebola?’ That may not be something that’s culturally acceptable’, a Johns Hopkins epidemiologist said on National Public Radio.” Frankfurter acknowledges that cultural differences may have contributed to the tension, but questions whether “more universally human processes” went unacknowledged: “In what culture *would* it be acceptable or productive to walk into a village and so brusquely identify and inform people that they only have days to live?” The framing of Ebola as an exoticised and racialised African problem engenders neglect and renders invisible the pain and suffering of individuals and communities affected [22]. 

Other varieties of ‘Us versus Them’ emerged during the outbreak, as, for example, in the mutual distrust that flared between some communities and government and public health authorities. Resistance to disease control measures was marked in all three countries, ranging from passive to violent conflict and explicit rejection of control measures [29,44]. Regional observers understood these actions within a landscape of historical and entrenched distrust and suspicion rooted in previous government interventions [19]. Ebola spread through countries “where war and limited post-conflict recovery still leave their legacy in impoverished infrastructure, capacities and discontent—fuelled by the failure of post-conflict recovery processes fully to address the questions of rights and employment that underlay the civil wars in the first place” [1]. Resistance in Liberia, for example, led to the dismantling of an Ebola screening unit by residents of the West Point District, as they viewed the unit as a risk to their safety. This action subsequently led to violent clashes between soldiers and protesters and to the quarantining of the whole area [29]. 

Reports of attacks by local communities on health workers and health care facilities “may give an impression of a violent, irrational people, resisting health care workers and government officials, whose only wish had been to aid an afflicted people” [45]. Indeed, the factors underlying violence towards and rejection of authorities responsible for implementing infection control went unexamined in reporting. Community resistance should, however, be analysed in context [45]. The fear and concern of the protesters was not unwarranted, given that when ambulances transported suspected infected individuals to Ebola Treatment Units (ETUs), health authorities rarely informed families as to where their relatives had been taken or if they had tested positive, survived or perished [45]. As regards the aforementioned quarantining measures, hundreds were forced to remain in their homes, without sufficient food or water, and a lack of basic sanitation and running water. Resistance was also reported against measures of surveillance, isolation and contact tracing. In Guinea, for example, Ebola contact tracing that traced the movements of individuals and documented friendships was experienced as “highly intrusive political surveillance from well-resourced state officials working with an intensity hitherto unknown and in regions where state authorities usually display little interest.” [28]. In response to resistance, the Liberian government made it illegal to hide an Ebola-infected patient, with the crime punishable by a prison sentence of two years [29]. Again, the heavy-handed response and insensitivity of government and health agencies served to further exacerbate already-heightened tensions and fears. It is important to note here that such resistance to infection control measures is not new or unique to Africa, as the historical record shows. For example, suspicion and violence towards health workers and health facilities was rife during the first pan-European cholera wave of the 1830s, with riots across the continent, and as far as Russia and across the Atlantic to America [44]. 

Resistance may also have occurred along colonial lines [1,19,44]. The international response was “starkly divided along colonial (and quasi-colonial lines)” [44]; France assumed responsibility for Guinea, the United States for Liberia and Britain for Sierra Leone. Wilkinson and Fairhead surmise that “Perhaps there was something qualitatively different in the styles of foreign assistance each country received through their ex-colonial power” [44]. For example, a “fraught decolonizing relationship between Guinea and France” [19] may have limited France’s response to the outbreak, with implications on the ground. 

### 2.4. Science Versus Other Practices 

The Ebola epidemic in West Africa has also highlighted the clash between biomedical systems and traditional medicines and practices [16,21,24]. Both systems exist in parallel in sub-Saharan African countries [16]. Much of Western Africa still depends heavily on traditional practices, with, for example, 70% of the population in Ghana relying solely on traditional medicine [40]. Traditional healers often have greater legitimacy than that of government health workers; their legitimacy derives from demonstrated compassion and an amalgamation of holistic approaches to health [21]. However, inhabitants of certain prefectures in the Forest Region of Guinea have long been familiar with visiting health posts, maternity clinics and hospitals as “part of the larger therapeutic landscape that embraces a variety of healing traditions” [28]. Moreover, Fairhead reports that local residents do not make sharp distinctions between “biomedical” and “traditional” health practices. Rather, other therapeutic distinctions are just as relevant; “For example, the difference between specialists who treat familiar ailments and those who treat unfamiliar ones; health care providers who treat predominantly men or women; or those who require upfront compensation or allow for deferred payment” [28]. Given the lack of treatment or an efficacious vaccine for Ebola, not to speak of almost non-existent health facilities, it is both unsurprising—and understandable—that many individuals relied on traditional healers for help [16]. 

At the same time, both popular and official (i.e., World Health Organisation (WHO) and Centre for Disease Control) accounts depicted African “beliefs” about disease aetiology and transmission as “ignorant and backwards” and “supposedly hindering or counteracting more enlightened epidemic control efforts” [13]. Western media depicted African culture—as opposed to a lack of basic health infrastructure and diagnostic capacity—as a major problem for containment [13,24]. Once more, culture was reframed as a “risk factor” for infection with respect to assumptions about African “Otherness” [13].

Specific cultural practices have not been unimportant [24]; this was particularly the case for local and regional practices of caring for the sick and in burial practices. However, western narratives omitted the overriding cultural and social considerations underpinning these practices. 

### 2.5. Cultural Beliefs and Practices 

Funeral and burial practices in the region are not simply concerned with the disposal of a body [46]. Such practices are “fundamental to the future of the deceased, their relatives, the wider community and the environment” [28]. The cultural and community importance of funerals is part of the process of “unmaking” a marriage at death and related decisions about land rights [39]. The need to maintain lineage rights, land tenure, and inter-family and inter-village relations helps to explain the “tenacity with which villagers defended funeral practices, despite official injunctions on unauthorized(*sic*) funerals” [39]. 

Due to the nature of its transmission, the Ebola virus is particularly influenced by cultural and behavioural practices that take place in the household and community levels and within a hospital setting [1,40]. Traditional burial practices, involving washing and touching of the deceased person, have been linked to 60% of Ebola cases in Guinea (40). Communities have been quick to learn and adapt practices to reduce transmission of the virus [1,39]. As communities in the three countries learned about the realities of Ebola, they began to undertake their own safe care, isolation and burial, just as communities that have longer experience with Ebola, e.g. DRC and Uganda, have done already for decades [1].

There is another set of local practices around farming, livelihoods and hunting, around which misleading narratives of human-environment relations have formed, with serious consequences for local communities. We turn to these practices next. 

### 2.6. Human-Ecological Dynamics 

Another set of tropes that include misleading assumptions and myths concerns the initial zoonotic “spillover” event. This storyline places ‘environmental blame’ on farmers who are deforesting a landscape and exposing themselves to bats for the first time [1,47]. The storyline goes as follows: “(…) poverty drives people to expand their range of activities, plunging deeper into the forest to expand the geographic, as well as species range of hunted game, and to find wood to make charcoal and deeper into mines to extract minerals, enhancing their risk of exposure to Ebola virus and other zoonotic pathogens in these remote corners” [48]. This story obscures the fact that “People and bats have long cohabited in this ancient, anthropogenic forest landscape with its mosaic forest, bush, and savannah, shaped by settlement and farming, war and trade, and everyday social and ecological life” [21]. Crucially, this particular storyline omits the actual primary drivers of environmental change in contemporary West Africa, namely expansive ‘land grabs’ designed to attract international investment in large-scale export-oriented mineral extraction, timber extraction and agribusiness activities, in particular, palm oil production [23]. The takeover of agricultural land by agribusiness has, in turn, led to massive ecological perturbations associated with ecological ‘phase shifts’ that result in enhanced risk of disease spillover events for rural communities [23,26]. Land grabs not only push people out of their homes and livelihoods, but also reconfigure ecological system dynamics and reduce biodiversity by causing fragmentation in habitats, including those of the wild bat [23]. Such ecological changes in the outbreak region may create the opportunity for direct exposure to infected bats, potentially creating transmission pathways [40,49]. 

Bushmeat hunting and consumption became particularly vilified as a hazardous practice in the discourse around the outbreak. [21,50]. While the handling and butchering of infected animals have been found to have a role in previous Ebola outbreaks, and particularly in the initial spillover event that generated the outbreak, epidemics are primarily driven by human-to-human transmission, as was the case here [51]. However, governments across the region implemented a ban on the hunting and consumption of meat derived from wild animals, with public health messages highlighting the infectious potential of ‘bushmeat’ [51]. Meat from wildlife has significant cultural, traditional, economic and medicinal value to indigenous people in tropical African rain forest regions [52]. Not only did the ban deprive people of a vital source of protein, as meat from ‘domestic animals’ is unaffordable for many, it also had negative implications for hunters and others engaged in the wild meat trade [51,52]. Crucially, this kind of public health communication led to a form of ‘cognitive violence’ [1] or ‘epistemic dissonance’ [51] between people’s practical and social experiences with bushmeat on the ground and the public health framing of bushmeat as hazardous [1,51,52]. 

We began with a discussion of the ‘outbreak narrative’ and conclude this section now with some thoughts regarding what is left remaining once the story is finished. Specifically, we turn to the framing of ‘lessons learned’, a final framing that can serve to mitigate, or conversely, exacerbate aspects and processes of marginalisation.

### 2.7. Reviewing ‘Lessons Learned’

As the ferocity of the 2014–2016 Ebola outbreak ebbed, a flow of publications—from academics and scholars, and from organisations such as the World Health Organisation (WHO), Médecins Sans Frontières (MSF), and a collaborative document from the United Nations, United Nations Development Programme (UNDP), World Bank, the European Union (EU) and the African Development Bank—emerged about the ‘lessons learned’ from the Ebola catastrophe [53]. There was much criticism of the tardy response of the WHO and widespread praise for MSF, but a general feeling that, with these lessons learned, things would be different next time [54]. Indeed, several commentators noted that there would be another outbreak, sooner rather than later and that indeed has happened. 

What *were* the lessons learned? They were all along similar themes: The need to strengthen fragile health systems; the need to improve surveillance and response capacities; the need to improve communication and to build trust within affected communities; the need for a coherent and coordinated global governance system; and the need to incentivise—and cultivate the political will for—the development of new vaccines and therapies for neglected diseases [53,54]. 

And yet here we are again, in the midst of the second-largest outbreak of Ebola—in the DRC—and a very recent warning from the WHO that big outbreaks of deadly diseases like Ebola “are the new normal” [55]. At the same time, as happened in the West Africa outbreak, the routing of funds for other health system activities in tackling the epidemics occurs at the expense of other health issues, such as measles, cholera and malaria [35,41]. 

So, what can we conclude from this state of affairs? Smith and Upshur (2015) have been critical of the ‘lessons learned’ terminology, as it suggests that the learning has already occurred [53]. Rather, they suggest that this initial exercise be framed as ‘lessons identified’ “which leaves outstanding the task of understanding and embodying those lessons, their implications and how they ought to be translated into actionable guidance and ultimately constructed or incorporated into institutional structures, policies and practices” [53]. Moreover, learning these lessons involves not only understanding and reflecting on the inadequacies of the response, but also on *why* these inadequacies exist, along with the factors underpinning and perpetuating these failures. 

Crucially, these lessons and themes are “steeped in ethics” [53], concerning values, value-conflicts and questions of what is morally right and wrong. However, the ethical character of the lessons and normative challenges inherent in responsively addressing our failures are largely absent in the “lessons learned” discourse [42,53]. The human rights elements—the basic human right to health—underlying these large outbreaks are also glaringly absent [56]. While attempts to improve the global outbreak responsive capacity are laudable (and indeed visible in responses to the current DRC outbreak) such stopgap solutions are not the answer [42,53,57,58,59]. We need a new moral orientation or, as put starkly by Caplan (2015), “Ethical business as usual in the face of an epidemic that kills 70% of its West African victims strikes many as ethical fiddling while Africa dies” [42]. Furthermore, we need to prioritise ‘quality as a cure’ [57] that is “care that is safe, effective, and delivered in ways that respect the dignity of individuals in the context of their own “local moral worlds” [57]. In tandem with the (re)construction of healthcare systems based on basic principles of quality assessment [57], it is important to be attentive to aspirations and concrete operationalisations of social justice. 

This paper is reaching its conclusions, its own ‘lessons learned’. We may not envision the one best way to govern these epidemics—be they conceived as ‘outbreaks’ or otherwise—but, as we are engaged in these collective experiments, the very least that ought to be done is to collectively learn from them. At the same time, while we strive to keep the learning open, we should always be wary of instrumentalising the ‘others’—i.e., the ‘othered’, be they individuals, groups, experimentees, at the margins—in favour of the collective.

## 3. Conclusions

This paper seeks to situate and explicate processes of marginalisation and their bearing on health challenges faced by marginalised people and populations, using the catastrophic West Africa Ebola outbreak as a paradigmatic example. The paper proceeds through a series of reframing moves, that is, each section comprises the identification of a mode of marginalisation and its unpacking. Marginalisation denotes processes, and it concerns relationships. We have endeavoured to unpack and illuminate practices, stories and histories, unequal distributions of power, knowledge and resources and the consolidation of inequalities through institutions. Moreover, we have emphasised the relational aspect of marginalisation in this particular case; between ‘Us and Them’ and north and south, between the past and the present, in the lived experiences of those directly affected by the outbreak and between diverse ways of knowing and understanding the world. Finally, we reflect on the ‘ought’, that is, the normative and ethical nature of framings of marginalisation and the need to pull them in from the margins to the centre. 
